# Training and Validating a Knowledge-Based Model for Intensity Modulated Proton Therapy of Prostate and Pelvic Lymph Nodes

**DOI:** 10.7759/cureus.83458

**Published:** 2025-05-04

**Authors:** Parker Anderson, Yihang Xu, Ricky Bui, Jonathan Cyriac, Robert Kaderka, Elizabeth Bossart, Nathan Hanson, Nesrin Dogan

**Affiliations:** 1 Radiation Oncology, University of Miami Miller School of Medicine, Miami, USA; 2 Radiation Oncology, University of Miami, Miami, USA; 3 Radiation Oncology, University of Miami Sylvester Comprehensive Cancer Center, Miami, USA

**Keywords:** automated treatment planning, intensity modulated proton therapy, knowledge based planning, pelvic lymph nodes, prostate cancer

## Abstract

Introduction

In this work, we aimed to create and assess the performance of a knowledge-based planning (KBP) model for optimizing intensity-modulated proton therapy (IMPT) in the treatment of prostate cancer involving pelvic lymph nodes (LNs).

Materials and methods

Fifty patients previously treated with IMPT to the prostate/prostate bed, including LNs and optional gross tumor volume (GTV) boost, were used for the training of a KBP model. The model was iteratively refined by replanning a subset of 20 of these patients. For validation, 20 patients not included in the model training set were used. Treatment plans were optimized using the objective list predicted by the model. Plan quality was evaluated using dosimetric metrics for both target and organs at risk (OARs), and the results were compared with manually generated plans using paired t-tests (p < 0.05).

Results

Eighteen out of 20 plans generated by the model were deemed to be clinically acceptable without the need for additional adjustments. The plans produced by the model demonstrated comparable robustness in clinical target volume (CTV) coverage. Significant improvements in OAR sparing were achieved for the rectum (V40Gy = -4.26 ± 3.00%), bladder (V40Gy = -6.36 ± 4.34%), and penile bulb (Dmean = -1.61 ± 9.76 Gy) when using the KBP model, compared to the manual plans. Other significant differences include slightly higher doses to the cauda equina (D0.03cc = 3.44 ± 6.09 Gy) and the left femur (D0.03cc = 2.50 ± 3.69 Gy) when compared to manual plans. No statistically significant differences were found for other OARs.

Conclusions

This study demonstrated that the KBP model produced plans comparable to manually generated clinical plans, and these plans are clinically acceptable. The iterative tuning process improved the quality of plans generated by the KBP model.

## Introduction

Radiation treatment planning methods currently rely on the time and expertise of the planner, as well as the success of repetitive trial-and-error optimization techniques. As plans can be created somewhat inconsistently, there has been a push for automated methods that can generate better and more consistent plans, regardless of the planner’s experience. This has led to the adoption of knowledge-based planning (KBP), a data-driven approach to intensity-modulated radiotherapy (IMRT) planning that learns from previously created high-quality plans. By utilizing these high-quality plans, a planning model is developed that can predict dose-volume histogram (DVH) and optimization objectives for new plans [1-5]. The objectivity inherent in the KBP process helps improve plan consistency and overall plan quality [6]. KBP methods are increasingly used in clinical settings for many treatment sites and have been shown to reduce optimization times and lower doses for organs at risk (OARs) [6-8].

KBP for intensity-modulated proton treatment (IMPT) has been developed to incorporate the unique physical characteristics of protons (e.g., no dose beyond the Bragg peak) into the DVH estimation model, showing promising clinical applications [9]. KBP is already being used in some clinics as the starting point for the planning process and has been shown to be comparable to manual planning, while also reducing optimization times [10,11]. These faster optimization times are particularly beneficial in adaptive radiotherapy (ART), where new plans must be generated within a limited timeframe [12,13]. Previous studies have demonstrated the use of KBP in IMPT for the treatment of prostate, head-and-neck, brain, liver, and gastroesophageal carcinomas [14-18].

A previous study investigated the implementation of KBP for IMPT of the prostate with pelvic lymph nodes (LNs) [13]. However, it only included a single dose/fractionation scheme and did not account for integrated boost or prostate bed cases. In clinical practice, it is common to prescribe multiple dose levels for IMPT of the prostate and prostate bed, which should be taken into account when developing a comprehensive KBP model. In this work, we developed and validated a KBP model that incorporates various prescriptions and dose levels for IMPT treatment of the prostate/prostate bed with pelvic LNs. Additionally, we describe the process of refining plans generated by the KBP model and compare the final plans with those created through manual optimization.

## Materials and methods

Patient cohort

This study, including the patients involved, was approved by the Institutional Review Board (IRB) under approval number MOD00016844. A total of 50 patients, previously treated with IMPT to either the prostate or the prostate bed with pelvic LNs (with and without a simultaneous integrated boost to the prostate or involved regional LNs), were used as a training set for this KBP model (see Table 1 and Table 2 for a list prescription and the number of patients per prescription type). A variety of dose prescriptions were employed to account for the potential variations in IMPT treatment for prostate cancer. For intact prostate cases, the dose ranges were 70.2-80 Gy to the prostate, 46.8-56 Gy to the LNs, and 65-88 Gy to the boost volumes. For prostate bed cases, the dose ranges were 57.5-68 Gy to the prostate bed, 49.5-52.7 Gy to the LNs, and 60-74.8 Gy to the boost volumes.

**Table 1:** Dose prescriptions to target volumes for intact prostate with pelvic lymph node cases CTV, clinical target volume; GTV, gross tumor volume; LN, lymph nodes; SV, seminal vesicle

CTV_Prostate (Gy)	CTV LN (Gy)	Lymph Nodule GTVp (Gy)	Primary Prostate Lesion GTVp (Gy)	Number of Patients
80	56	-	-	2
80	56	74	-	1
80	56	72	-	1
80	56	-	86	15
80	56	-	88	9
80	56	72	86	2
80	56	76	88	1
80	56	72	88	1
70.2	46.8	65	-	1
70.2	56	-	74.1	1

**Table 2:** Dose prescriptions to target volumes for prostate bed with pelvic lymph node cases CTV, clinical target volume; GTV, gross tumor volume; LN, lymph nodes; SV, seminal vesicle

CTV_Prostate (Prostate Bed) (Gy)	CTV LN (Gy)	Lymph Nodule GTVp (Gy)	Number of Patients
68	52.7	-	4
68	52.7	74.8	5
68	52.7	73.1	5
66	49.5	69.3	1
57.5	50	60	1

Planning

Manually generated IMPT plans used robust optimization (Eclipse 16.1, Varian Medical Systems, Palo Alto, CA) for clinical target volumes (CTVs) to account for the high sensitivity of IMPT to uncertainties [19-21]. Robust optimization was performed with either 3- or 5-mm setup uncertainties (as specified by the physician) in all directions, along with ±3.5% proton range uncertainties, resulting in 12 different uncertainty scenarios. The worst-case scenario was required to achieve V95% > 95% (95% of the volume receiving more than 95% of the prescription dose) for all CTVs. For each patient, IMPT plans were generated using multifield optimization (MFO), with three to four fields employed depending on the target location and anatomy, typically two opposed lateral fields and one posterior-anterior field. The non-linear universal proton optimizer (NUPO 16.1, Eclipse, Varian Medical Systems, Palo Alto, CA) was utilized for optimization, while the proton convolution superposition algorithm (PCS 16.1, Eclipse, Varian Medical Systems, Palo Alto, CA) was utilized for dose calculation. The dose constraints for the targets and OARs are listed in Table 3. Most plans were expected to meet target coverage requirements as well as all OAR constraints listed in Table 3. However, in some cases where both target coverage and OAR sparing objectives could not be achieved simultaneously, physicians made case-by-case decisions, prioritizing one over the other based on clinical judgment.

**Table 3:** CTV and OAR dose constraints for the IMPT plans V100 and V95 represents relative volume receiving more than 100% and 95% of the prescription dose respectively; Dmax represents the maximum absolute or relative dose delivered to the structure; Dmean represents the mean absolute dose delivered to the structure; D0.03cc represents the dose received by 0.03cc of the structure; V40Gy, V65Gy, and V75Gy represent the relative volume of the structure receiving >40 Gy, 65 Gy, and 75 Gy, respectively. CTV, clinical target volume; GTV, gross tumor volume; LN, lymph nodes; OAR, organ at risk; IMPT, intensity-modulated proton therapy; SV, seminal vesicle

Structures	Clinical Constraints
CTV	Dmax < 115%
GTV	V100 > 95%
CTV Prostate	V100 > 95%
CTV Prostate	V95 (Worst-Case Scenario) > 95%
CTV Distal SV	V100 > 95%
CTV Distal SV	V95 (Worst-Case Scenario) > 95%
CTV LN	V100 (%) > 95%
CTV LN	V95 (Worst-Case Scenario) > 95%
Rectum	V40Gy < 35%
Rectum	V65Gy < 17%
Rectum	V75Gy < 10%
Bladder	V40Gy < 50%
Bladder	V65Gy < 25%
Bladder	V75Gy < 15%
Bowel Bag	V40Gy < 150cc
Bowel Bag	D0.03cc < 70Gy
Left Femur	D0.03cc < 50Gy
Right Femur	D0.03cc < 50Gy
Cauda Equina	D0.03cc < 50Gy
Penile Bulb	Dmean < 50Gy

Model description

The KBP model was created using plans from 50 prostate cancer patients previously treated with IMPT and the commercial KBP solution RapidPlan PT (ver. 16.1, Varian Medical Systems, Palo Alto, CA). During model creation, optimization objectives for each volume were either automatically set or manually defined by the user, serving as a baseline for the plans generated by the model. These optimization objectives have been shown to significantly impact the outcome of plans produced by KBP models [11]. A 1-cm ring structure was created around the target volume to minimize hotspot spillage outside the targets. Overlapping dose levels were managed by using target-specific optimization volumes (OVs) to better control the maximum dose within each volume. Lower-dose targets were cropped from higher-dose targets based on a 5% per millimeter fall-off rule. Robust optimization was applied to all CTVs, and an upper objective was set for femoral head volume. All model-generated plans were optimized using the model-generated objective list without any manual adjustments.

Iterative tuning

To evaluate the outcomes of plans generated by this model, a subset of 20 training patients was used. Plans were created using the KBP model, employing the same beam angles and uncertainty scenarios as the manual plans. The plans were optimized using the objective list generated by the KBP model, without any further adjustment. All plans were normalized to ensure that the dose constraints for all targets, including the worst-case scenario for all CTVs, were satisfied. The DVHs from these plans were then compared to the clinical plans used for actual patient treatment. The clinical plans, created through manual optimization, served as the benchmark for clinical quality. The tuning process followed a trial-and-error approach, where plans were generated by the model and compared to the manually optimized plans. If the model-generated plans were substantially worse in any aspect, the optimization objectives of the model were adjusted, and the process was repeated until all plans met desired criteria. Our goal was to generate plans that were at least clinically comparable to the manual plans, if not better. Data analysis was performed to ensure that there were no statistically significant differences or that the plans generated by the KBP model were not worse, in terms of the evaluated dose-volume indices. Once these goals were met for all plans in the tuning set, model validation was carried out. Table 4 provides the complete list of objectives used in the model after tuning. 

**Table 4:** Final optimization objectives * indicates robust optimization was enabled for these objectives. CTV, clinical target volume; GTV, gross tumor volume; gEUD, generalized equivalent uniform dose; L, left; LN, lymph nodes; OV, optimization volume; R, right; SV, seminal vesicle

Structure	Objective Type	Relative Volume (%)	Dose	Priority
CTV Prostate	Lower	99.9	101.0%	175
	Lower*	98.0	99.0%	210
	Lower gEUD	-	100.0	200
	Lower	99.9	102.0%	175
CTV LN	Lower*	98.0	99.0%	210
	Lower gEUD	-	100.0%	200
	Lower	99.9	101.0%	150
CTV SVs Dist	Lower*	98.0	99.0%	175
	Lower gEUD	-	100.0%	200
	Upper	0.0	101.0%	175
GTVp	Lower	97.0	101.0%	200
	Lower	100.0	97.0%	175
	Upper	0.0	105.0%	250
OV_LN	Upper	0.0	105.0%	250
OV_Prostate	Upper	15.0	87.0%	50
Bladder	Upper	25.0	75.0%	50
	Upper	50.0	44.0%	50
	Upper (Fixed Vol., Generated Dose)	0.0	Generated	119
	Line (Pref. Target)	Generated	Generated	140
	Upper	0.0	56.00 Gy	200
Bowel Bag	Upper	1.0	40.00 Gy	120
	Upper (Fixed Vol., Generated Dose)	0.0	Generated	99
	Mean	-	Generated	99
	Line (Pref. Target)	Generated	Generated	150
	Upper	0.0	66.0%	200
Cauda Equina	Upper (Fixed Vol., Generated Dose)	0.0	Generated	99
	Line (Pref. Target)	Generated	Generated	100
	Upper*	0.0	37.00 Gy	200
Femoral Head Neck_L	Upper (Fixed Vol., Generated Dose)	0.0	Generated	150
	Mean	-	Generated	99
	Line (Pref. Target)	Generated	Generated	100
	Upper*	0.0	37.00 Gy	200
Femoral Head Neck_R	Upper (Fixed Vol., Generated Dose)	0.0	Generated	150
	Mean	-	Generated	99
	Line (Pref. Target)	Generated	Generated	100
	Mean	-	2.00 Gy	100
Kidney_L	Mean	-	2.00 Gy	100
Kidney_R	Upper	0.0	80.0%	100
Penile Bulb	Upper (Fixed Vol., Generated Dose)	0.0	Generated	99
	Mean	-	25.00 Gy	100
	Line (Pref. Target)	Generated	Generated	100
	Upper	5.0	87.0%	200
Rectum	Upper	15.0	75.0%	200
	Upper	30.0	50.0%	200
	Upper	0.0	100.0%	200
	Line (Pref. Target)	Generated	Generated	140
	Upper	0.0	88.25 Gy	150
Ring_Bst	Upper (Fixed Vol., Generated Dose)	0.0	Generated	250
	Line (Pref. Target)	Generated	Generated	100
	Upper	0.0	56.25 Gy	150
Ring_LN	Upper (Fixed Vol., Generated Dose)	0.0	Generated	250
	Line (Pref. Target)	Generated	Generated	100
	Upper	0.0	80.25 Gy	150
Ring_Prostate	Upper (Fixed Vol., Generated Dose)	0.0	Generated	250
	Line (Pref. Target)	Generated	Generated	100
	Upper	0.0	30.0%	100
Skin	Upper	0.0	50.00 Gy	100
	Upper (Fixed Vol., Generated Dose)	0.0	Generated	99
	Line (Pref. Target)	Generated	Generated	50
	Upper	0.0	37.00 Gy	250
Spinal Cord	Upper	0.0	25.00 Gy	50

Model validation

A separate set of 20 patients, not included in the training cohort, was used for validation. Among these, 11 were prostate patients with prostate gross tumor volume (GTV) boosts, and nine were prostate bed patients, seven of whom also received pelvic LN boosts. These 20 plans were re-planned using the tuned KBP model, and the DVH parameters were compared to those of the manually optimized plan. Target volume coverage and OAR sparing from the KBP plans were compared to those from manually optimized plans, including worst-case scenarios (the scenarios with greatest possible uncertainty) for target volumes at V95%. Differences in DVH parameters between the manually optimized IMPT plans and KBP-generated plans were assessed using a paired t-test (p < 0.05).

## Results

Table 5 shows a comparison of the dose-volume indices between model-created plans and manually optimized plans for the 20 validation patients. In the validation group, KBP model plans achieved significantly higher V100% (2.34 ± 1.91 Gy) for the GTV boost volume as compared to the manual plans. There was no statistically significant difference in V95% of the worst-case scenario for the CTVs between KBP model-generated plans and manual plans, indicating comparable robustness of CTV coverage. OAR sparing was significantly better for the rectum and bladder across all dose-volume indices, except for bladder V65Gy (Table 5). Additionally, there was a significant reduction in the dose to the penile bulb (Dmean), with KBP plans showing a decrease of -11.61 ± 9.76 Gy. KBP-generated plans exhibit slightly higher D0.03cc for the left femoral head (2.50 ± 3.69 Gy) and cauda equina (3.44 ± 6.09 Gy). However, these small increases in dose remained well below clinical constraints and are unlikely to have clinical significance. This slight increase in dose was not observed during our iterative tuning process: had it been, further adjustments would have been made. It is likely that the model prioritized the sparing of higher-risk OARs, allowing a small trade-off in dose to these lower-priority volumes.

**Table 5:** Aggregate comparison of KBP-generated plans to manually optimized clinical plans Comparison of dose-volume indices between manually optimized clinical plans (clinical) and knowledge-based planning generated plans for 20 validation patients using a paired t-test. A p-value < 0.05 indicates a statistically significant difference. V100 and V95 represent relative volume receiving more than 100% and 95% of the prescription dose, respectively; Dmax represents the maximum absolute or relative dose delivered to the structure; Dmean represents the mean absolute dose delivered to the structure; D0.03cc represents the dose received by 0.03cc of the structure; V40Gy, V65Gy, and V75Gy represent the relative volume of the structure receiving >40 Gy, 65 Gy, and 75 Gy, respectively. CTV, clinical target volume; GTV, gross tumor volume; KBP, knowledge-based planning; LN, lymph nodes; SV, seminal vesicle

	Clinical	KBP	KBP - Clinical	t-value	P-value
CTV Dmax (%)	104.85 ± 1.69	104.95 ± 2.9	0.11 ± 3.05	0.16	0.87
GTV Prostate V100 (%)	96.57 ± 2.02	98.91 ± 1.76	2.34 ± 1.91	5.05	< 0.01
CTV Prostate V100 (%)	99 ± 1.56	99.15 ± 1.39	0.14 ± 1.6	0.41	0.69
CTV Prostate V95 (Worst-Case Scenario) (%)	97.39 ± 2.79	96.78 ± 1.27	-0.61 ± 2.78	-0.98	0.34
CTV Distal SV V100 (%)	99.99 ± 0.03	99.97 ± 0.07	-0.02 ± 0.08	0.60	0.60
CTV Distal SV V95 (Worst-Case Scenario) (%)	98.34 ± 0.65	98.25 ± 1.59	-0.09 ± 2.17	-0.11	0.91
CTV LN V100 (%)	97.75 ± 1.54	96.65 ± 1.43	-1.1 ± 2.71	-1.81	0.09
CTV LN V95 (Worst-Case Scenario) (%)	96.07 ± 1.88	96.9 ± 0.91	0.82 ± 1.95	1.89	0.07
Rectum V40Gy (%)	18.83 ± 5.68	14.57 ± 6.52	-4.26 ± 3	-6.34	< 0.01
Rectum V65Gy (%)	6.94 ± 2.85	4.33 ± 2.1	-2.61 ± 1.85	-6.32	< 0.01
Rectum V75Gy (%)	2.29 ± 2.48	1.28 ± 1.37	-1.01 ± 1.26	-3.60	< 0.01
Bladder V40Gy (%)	32.09 ± 19.35	25.73 ± 20.24	-6.36 ± 4.34	-6.56	< 0.01
Bladder V65Gy (%)	17.81 ± 18.43	15.63 ± 19.23	-2.18 ± 5.31	-1.84	0.08
Bladder V75Gy (%)	2.98 ± 3.6	1.65 ± 2.1	-1.32 ± 1.68	-3.52	< 0.01
Bowel Bag V40Gy (cc)	114.71 ± 31.73	123.46 ± 39.39	8.75 ± 27.3	1.43	0.17
Bowel Bag D0.03cc (Gy)	60.16 ± 5.37	61.56 ± 4.77	1.4 ± 5.18	1.21	0.24
Left Femur D0.03cc (Gy)	35.99 ± 6.18	38.49 ± 5.74	2.5 ± 3.69	3.03	0.01
Right Femur D0.03cc (Gy)	37.31 ± 4.14	38.62 ± 6.26	1.31 ± 6.23	0.94	0.36
Cauda Equina D0.03cc (Gy)	15.57 ± 4.85	19.01 ± 7.62	3.44 ± 6.09	2.46	0.02
Penile Bulb Dmean (Gy)	24.13 ± 17.81	12.52 ± 8.55	-11.61 ± 9.76	-5.32	< 0.01

**Figure 1:**
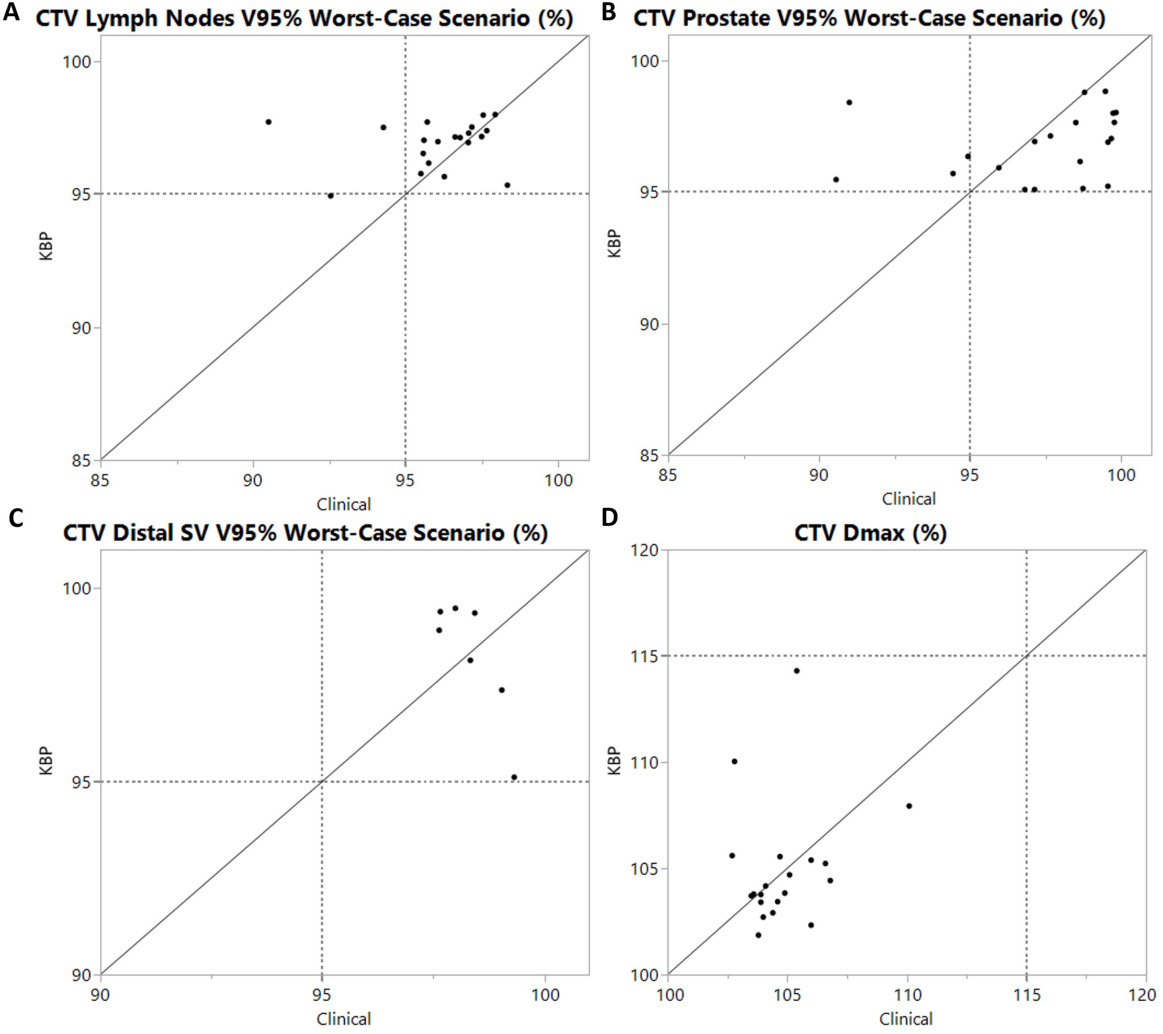
CTV parameters where each point is one plan with the x-coordinate being the manual clinical plan and the y-coordinate the knowledge-based planning (KBP)-generated plan value Points above the equality line indicate a higher value of the model-generated plan compared to the clinical plan and vice versa. The gray dashed line represents the value of dose-volume used in our institution. Subplots are labeled (A-D) on the left side of each panel: (A) CTV lymph nodes V95% in the worst-case scenario (%), (B) CTV prostate V95% in the worst-case scenario (%), (C) CTV distal seminal vesicle (SV) V95% in the worst-case scenario (%), (D) CTV Dmax (%) V100 and V95 represent relative volume receiving more than 100% and 95% of the prescription dose, respectively; Dmax represents the maximum absolute or relative dose delivered to the structure. CTV, clinical target volume

**Figure 2:**
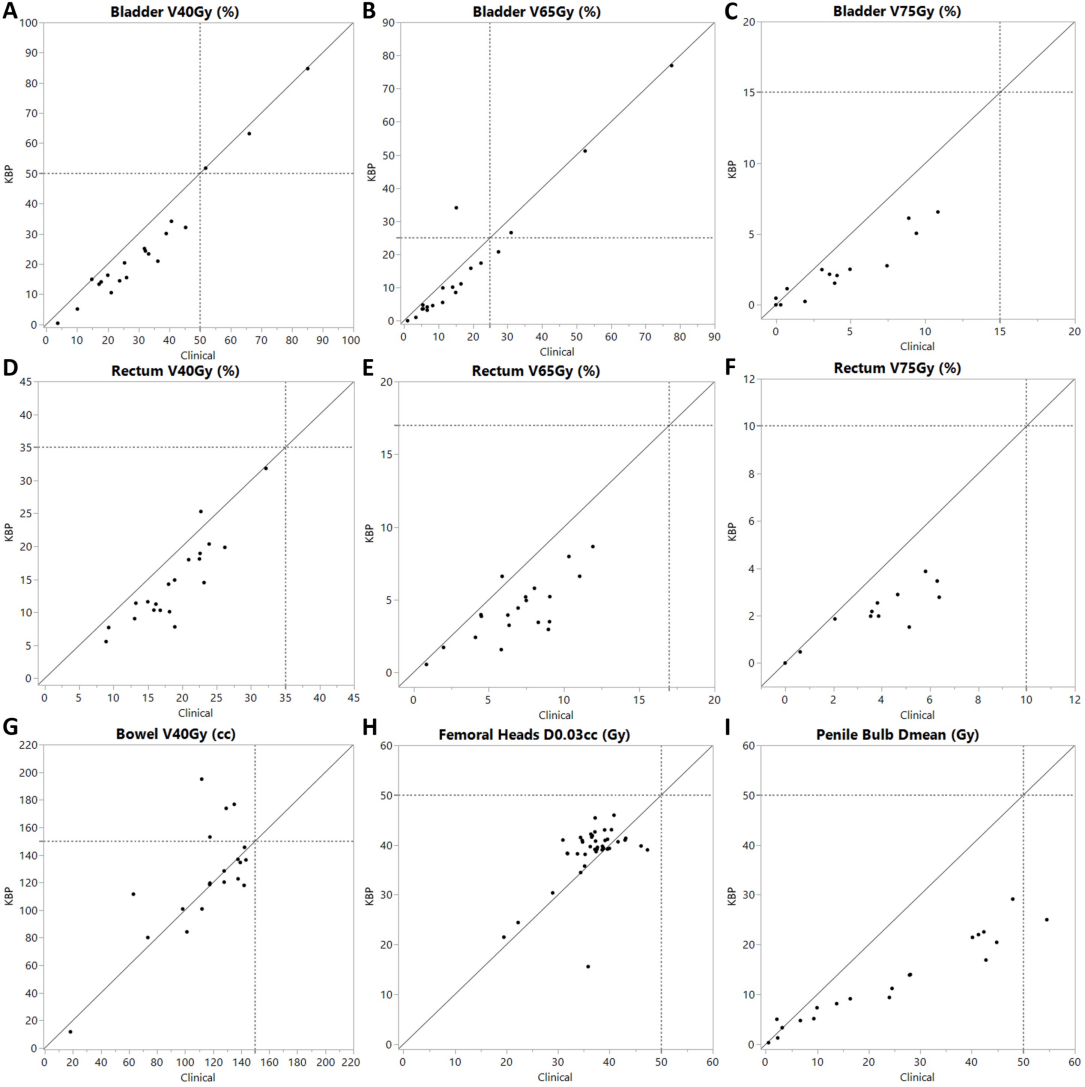
Organ at risk (OAR) parameters, where each point is one patient, with the x-coordinate being the clinical plan value and the y-coordinate the knowledge-based planning (KBP)-generated plan value The gray dashed line represents the value of dose-volume constraints used in our institution. Subplots are labeled (A-I) on the left side of each panel: (A) bladder V40Gy (%), (B) bladder V65Gy (%), (C) bladder V75Gy (%), (D) rectum V40Gy (%), (E) rectum V65Gy (%), (F) rectum V75Gy (%), (G) bowel V40Gy (cc), (H) femoral heads D0.03cc (Gy), (I) penile bulb Dmean (Gy). Dmean represents the mean absolute dose delivered to the structure; D0.03cc represents the dose received by 0.03cc of the structure; V40Gy, V65Gy, and V75Gy represent the relative volume of the structure receiving >40 Gy, 65 Gy, and 75 Gy, respectively.

Figure 1 and Figure 2 provide a case-by-case comparison of CTV coverage robustness and OAR sparing between model-generated and manually optimized plans. All model-generated plans achieved V95% > 95% for the worst-case scenario of CTV coverage. In contrast, three manual plans did not meet this criterion for CTV LN, and four manual plans fell short for CTV prostate, as a result of prioritizing bladder or bowel bag sparing over CTV coverage compared to the model-generated plans. For OAR sparing, one model-generated plan did not meet the bladder V65Gy constraint, whereas the manual plan did. However, this was achieved at the cost of reduced CTV coverage robustness in the manual plan. For the bowel bag, the model-generated plans failed to meet the clinical constraint in two cases, while the manual plans succeeded without compromising CTV coverage robustness.

## Discussion

The primary goal of this study was to develop a KBP model capable of generating plans that are clinically comparable to those created manually for IMPT treatment of the prostate or prostate bed with pelvic LNs. All cases used during the iterative tuning phase successfully produced plans that met this criterion. In the validation cohort, 18 out of 20 validation plans created with the KBP model were deemed clinically comparable to their manually optimized counterparts. The two cases from the validation group failed to meet the bowel bag constraint (V40Gy < 150cc), whereas the manually optimized plans succeeded in meeting this constraint without compromising the robustness of CTV coverage. In our iterative tuning process, all 20 tuning cases were adjusted to achieve bowel bag sparing that was at least comparable to manually optimized plans. Therefore, the two validation cases that did not meet the bowel bag constraint are considered outliers. This discrepancy may be due to the large bowel bag volume in those cases, which may have made it difficult for the model to accurately predict the achievable DVH curve. After manually increasing the priority of the bowel bag optimization objectives, we were able to produce plans to meet all clinical constraints. This demonstrates that while the KBP model provides a robust baseline for plan creation, a small number of cases may still require manual adjustments to ensure they are fully clinically acceptable.

One of the few other studies assessing the performance of a KBP model for IMPT of the prostate with pelvic LNs reported results similar to ours [13]. Their model, which was limited to patients receiving the same prescription, also produced plans that were clinically comparable to manually optimized ones. However, the only significant difference observed was slightly better sparing of the cauda equina volume in the model-generated plans compared to the manual plans. In comparison, our model showed slightly higher doses to the cauda equina and left femoral head, though these doses remained well within clinical tolerance. Notably, our model achieved significantly better sparing of the rectum, bladder, and penile bulb, as well as improved target coverage for the boost GTVs. These improved results may be attributed to our use of an iterative tuning process, which was not employed in the previous study. Previous studies have also demonstrated the efficacy of KBP methods for IMPT treatment of the prostate, but these studies were limited to patients with localized prostate cancer [16]. In this study, we incorporated a broader patient cohort, including those receiving prostate and prostate bed treatments with or without boost doses, across a variety of prescriptions and treatment volumes for the pelvic LNs. Our findings demonstrate that the KBP model effectively accommodates diverse clinical scenarios and treatment schemes, generating robust and high-quality treatment plans. The inclusion of multiple prescription types may require an additional tuning process to optimize outcomes across various treatment schemes. Notably, our tuning approach successfully generated robust plans that were at least comparable to clinical plans, requiring minimal further intervention, regardless of treatment complexity.

Each KBP-generated plan took approximately 20 minutes from optimization to final dose calculation, significantly faster than the two to four hours typically needed for manual plan creation by an expert. Moreover, the process of generating the plans is entirely passive with the use of the KBP model, which can significantly lighten the workload of the planner. By using a KBP model to generate an initial clinically acceptable plan, planners can focus more on addressing the specific challenges inherent to each case. While KBP can serve as a solid baseline for creating IMPT plans, it cannot fully replicate the nuanced judgment of an expert planner. Although most of our KBP-generated plans were clinically acceptable, they still require thorough evaluation by an expert planner to ensure high-quality dosimetry.

A potential limitation of this study is the uneven distribution of patient cases and treatment schemes in the training cohort. Specifically, there were only five cases with both prostate GTV boost and LN boost, which may limit the model's reliability for such scenarios. While the inclusion of a substantial number of cases with either prostate GTV boost or LN boost partially addresses this limitation, it remains unclear whether the model can consistently generate clinically acceptable plans for these specific prescription combinations. Nevertheless, the validation results, which encompass a variety of treatment schemes, demonstrate the model’s overall robustness. Furthermore, a previous study on photon-based KBP models has shown that increasing the training dataset size (e.g., from 33 to 66 to 97 cases) did not significantly impact plan quality for prostate treatments [22]. Therefore, the inclusion of 50 cases with diverse prescriptions in this study provides a reasonable and sufficient foundation for model development. Another limitation is that the training and validation data were derived from a single institution, reflecting its specific clinical practices, which may affect the model's application to other institutions. Delaney et al. demonstrated that a KBP model developed at a single institution could still efficiently generate head and neck IMPT plans of comparable quality to manually created plans from other centers with differing planning goals, although occasional higher KBP OAR doses were observed [23]. This suggests that with appropriate validation, single-institution models may hold promise for broader clinical implementation.

The future direction of this work involves exploring the broader clinical applications of the KBP model, beyond initial plan generation. By generating the DVH estimation and fast planning, the model could support comparative plan evaluation, aiding clinical decisions on modality selection based on dosimetric advantages [24,25]. Another important application is in the context of adaptive proton therapy, where rapid re-optimization is critical. The demonstrated efficiency and robustness of our KBP model suggest that it could serve as a powerful engine for generating adaptive plans within clinically feasible timeframes [12,13]. Additionally, the model can contribute to the quality assurance (QA) of manual planning by identifying deviations from dosimetric outcomes from the DVH estimation model, thereby flagging plans for further review. Previous studies have shown a strong correlation between predicted and achieved doses, supporting the feasibility of using KBP-based DVH predictions alone for automated, individualized plan QA in photon head and neck and prostate treatments [26,27].

## Conclusions

This study evaluated the use of KBP for IMPT in the treatment of prostate cancer with pelvic LNs. Through an iterative tuning process, the model generated plans that were clinically comparable to manual plans in target coverage and OAR sparing. Notably, the KBP model achieved improved sparing for the rectum, bladder, and penile bulb, along with better coverage of boost volumes. While most of the KBP-generated plans met clinical standards, a few required minor manual adjustments. Overall, the KBP model shows promise as a strong starting point for IMPT planning, offering potential time savings and improved planning efficiency. Further external validation across institutions and patient populations will be important to confirm the generalizability and robustness of the model.
